# A semi‐automated ASC speck assay to evaluate pyrin inflammasome activation

**DOI:** 10.1002/cti2.70054

**Published:** 2025-10-23

**Authors:** Pei Dai, Oliver Skinner, Xufeng Lin, Aiden Telfser, Stephanie Ruiz‐Diaz, Rohit G Saldanha, Katie Frith, Ming‐Wei Lin, Kahn Preece, Paul E Gray, Alberto Pinzon‐Charry, Anna Sullivan, Stephen Adelstein, Winnie WY Tong, Matthew JS Parker, Laila Girgis, Brynn Wainstein, Samar Ojami, Elissa K Deenick, Leonard D Goldstein, Michael J Rogers, Tri Giang Phan

**Affiliations:** ^1^ Precision Immunology Program Garvan Institute of Medical Research Sydney NSW Australia; ^2^ St Vincent's Healthcare Clinical Campus, School of Clinical Medicine, Faculty of Medicine and Health UNSW Sydney Sydney NSW Australia; ^3^ Department of Immunology Nepean Hospital Sydney NSW Australia; ^4^ Immune Biotherapies Program Garvan Institute of Medical Research Sydney NSW Australia; ^5^ Department of Microbiology and Immunology The University of Melbourne at The Peter Doherty Institute for Infection and Immunity Melbourne VIC Australia; ^6^ Computational Biology Group, Data Science Platform Garvan Institute of Medical Research Sydney NSW Australia; ^7^ Department of General Paediatrics Sydney Children's Hospital Sydney NSW Australia; ^8^ Department of Paediatrics Bankstown‐Lidcombe Hospital Sydney NSW Australia; ^9^ Randwick Clinical Campus, School of Clinical Medicine, Faculty of Medicine and Health UNSW Sydney Sydney NSW Australia; ^10^ Department of Immunology Sydney Children's Hospital Sydney NSW Australia; ^11^ Department of Clinical Immunology Westmead Hospital Sydney NSW Australia; ^12^ Department of Immunopathology, ICPMR Westmead Hospital Sydney NSW Australia; ^13^ Centre of Immunology and Allergy Research Westmead Institute of Medical Research Sydney NSW Australia; ^14^ Western Clinical School, Faculty of Medicine and Health University of Sydney Sydney NSW Australia; ^15^ Paediatric Immunology Department John Hunter Children's Hospital Newcastle NSW Australia; ^16^ Western Sydney University School of Medicine Sydney NSW Australia; ^17^ Queensland Children's Hospital Brisbane QLD Australia; ^18^ Department of Clinical Immunology and Allergy and Institute of Academic Medicine Royal Prince Alfred Hospital Sydney NSW Australia; ^19^ Central Clinical School, Faculty of Medicine and Health University of Sydney Sydney NSW Australia; ^20^ HIV and Immunology Department St Vincent's Hospital Sydney NSW Australia; ^21^ Department of Rheumatology Royal Prince Alfred Hospital Sydney NSW Australia; ^22^ Department of Rheumatology St Vincent's Hospital Sydney NSW Australia; ^23^ Monash Pathology, Monash Health Melbourne VIC Australia; ^24^ Monash Infectious Diseases, Monash Health Melbourne VIC Australia; ^25^ Department of Medicine Monash University Melbourne VIC Australia; ^26^ Kirby Institute Faculty of Medicine and Health, UNSW Sydney Sydney NSW Australia

**Keywords:** ASC speck, functional testing, inflammasome, *MEFV*, pyrin, VUS

## Abstract

**Objective:**

To develop a rapid functional assay to validate variants of uncertain significance (VUS) in the *MEFV* gene.

**Methods:**

Overactivity of the pyrin inflammasome pathway and ASC speck oligomerisation in response to stimulation with low concentrations of *Clostridium difficile* toxin A was directly visualised by immunofluorescence microscopy. A semi‐automated algorithm was developed to count cells and ASC specks.

**Results:**

The semi‐automated ASC speck assay is able to discriminate between healthy controls and patients with familial Mediterranean fever (FMF) and pyrin inflammasome overactivity with high sensitivity. It is also able to discriminate pyrin inflammasome overactivity from other autoinflammatory disease controls with high specificity.

**Conclusion:**

The semi‐automated ASC speck assay may be a useful test to functionally validate VUS in the *MEFV* gene and screen for pyrin inflammasome overactivity.

## Graphical abstract

A semi‐automated ASC speck assay using machine learning is able to discriminate between healthy controls and patients with familial Mediterranean fever (FMF) with high sensitivity. It is also able to discriminate FMF from other autoinflammatory diseases with high specificity.

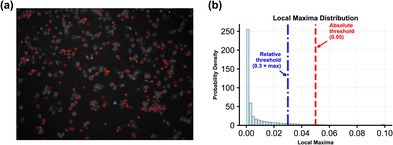

## INTRODUCTION

Familial Mediterranean fever (FMF) is an autosomal recessive autoinflammatory disease arising from biallelic gain‐of‐function (GOF) mutations within the *MEFV* gene that encodes the protein pyrin.^1^ Pyrin senses bacterial toxin‐mediated inhibition of RhoA GTPase and assembles the inflammasome via the adaptor apoptosis‐associated speck‐like protein containing a CARD (ASC) to cleave procaspase‐1 into active caspase‐1, leading to the cleavage and secretion of IL‐1β and IL‐18.^2^, ^3^ There are currently > 350 variants of uncertain significance (VUS) in the *MEFV* gene reported in the Infevers database (https://infevers.umai‐montpellier.fr/web/). Functional assays are therefore needed to determine the pathogenicity of *MEFV* variants, particularly in the heterozygous state.

Several assays of pyrin inflammasome activation have been developed for research purposes. These involve *in vitro* cell culture with clostridial toxins^4^, ^5^ or serine–threonine kinase PKN1/PKN2 inhibitors^6^ to inactivate RhoA GTPase, followed by measurement of the resulting cytokine secretion and pyroptotic cell death. However, these assays often use cell lines transfected with *MEFV* variants, are time‐consuming and require molecular biology and tissue culture expertise to perform. They also lack specificity, requiring some investigators to add colchicine to the culture conditions to distinguish healthy from pathogenic inflammasome activation by clostridial toxins.^7^, ^8^

A more direct method of measuring pyrin activation involves detecting ASC protein oligomerisation. In the steady state, ASC protein monomers are present diffusely throughout the cytoplasm. Inflammasome activation causes their oligomerisation into a single discrete supramolecular complex.^9^ These changes in the intensity and subcellular distribution of ASC staining can be inferred by analysing the ASC‐width to ASC‐area ratio using flow cytometry.^10^, ^11^, ^12^ Alternatively, immunofluorescence microscopy can directly visualise ASC speck formation in cell lines.^13^, ^14^ It remains unclear whether this approach can be used in primary patient cells to discriminate physiological from pathological inflammasome activation, or to differentiate FMF from other autoinflammatory diseases. Furthermore, manual counting of ASC specks by immunofluorescence microscopy is subjective, labour‐intensive and time‐consuming. Here, we describe a batched assay for rapid semi‐automated counting of ASC speck formation using frozen peripheral blood mononuclear cells (PBMCs).

## RESULTS

### Validation of semi‐automated ASC speck and cell counting

**Figure 1:**
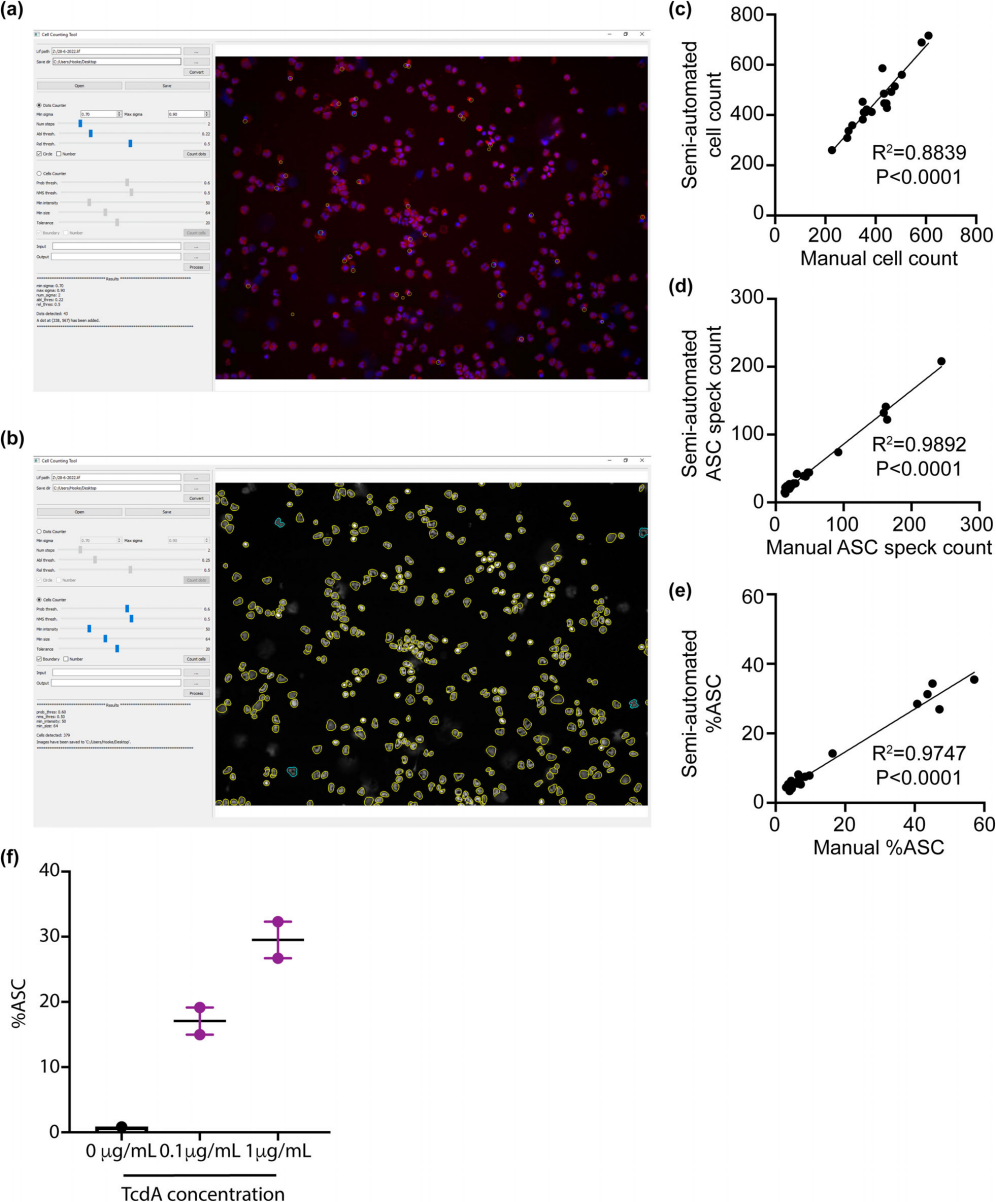
**(a)** Graphical user interface (GUI) for counting ASC specks. **(b)** GUI for counting cells. **(c)** Comparison of semi‐automated and manual cell counting. **(d)** Comparison of semi‐automated and manual ASC speck counting. **(e)** Comparison of semi‐automated and manual %ASC. **(f)** Comparison of zero, low (0.1 μg mL^−1^) and high concentration (1 μg mL^−1^) TcdA stimulation in healthy control samples.

A graphical user interface (GUI) programme was developed to allow rapid and objective live cell and ASC speck counting (Figure 1a and b). We validated the accuracy of the semi‐automated ASC speck and cell counting software by manually counting five separate images in cells from untreated healthy control, untreated disease control with homozygous M694V variant, healthy control incubated with 0.1 μg TcdA and disease control incubated with 0.1 μg TcdA. We found a significant correlation between manual semi‐automated counting of cells, ASC specks and final %ASC Speck (Figure 1c–e), confirming its validity. The software was used for all subsequent counting.

### ASC speck formation with low dose TcdA has high sensitivity for FMF

**Figure 2:**
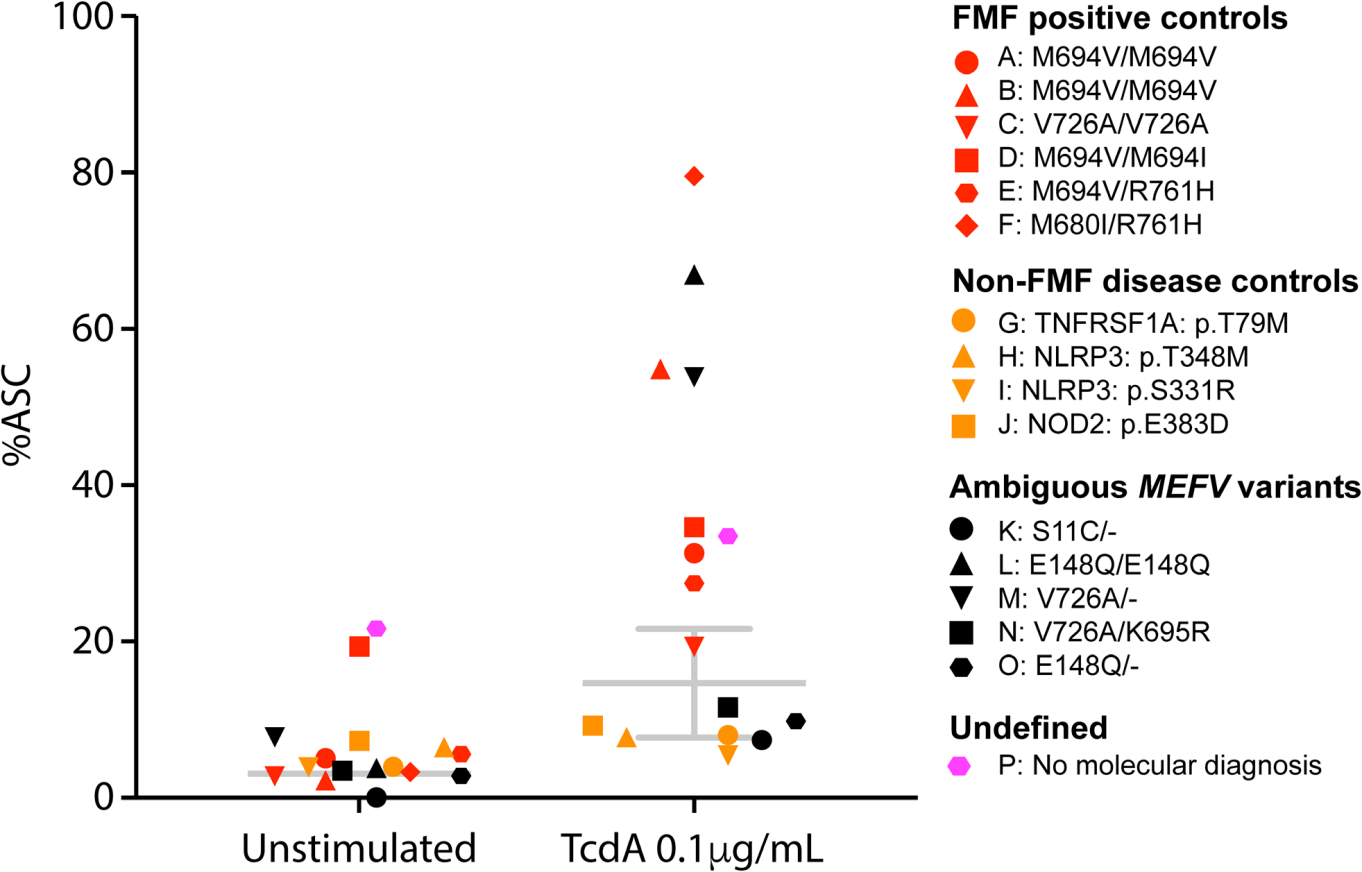
%ASC speck in FMF‐positive controls, non‐FMF disease positive controls and patients with ambiguous *MEFV* variants and patients with no genetic diagnosis. The reference range (RR) from healthy controls is shown in grey bars as the mean ± 95% CI.

We initially titrated the concentration of TcdA and observed dose‐dependent ASC speck formation in healthy control samples (Figure 1f). The low TcdA dose was chosen as a possible discriminator between physiological and pathological pyrin activation. Unstimulated healthy controls had a mean %ASC of 3.1 (95% CI: 1.4–4.8), and this increased to a mean of 14.7 (95% CI: 7.8–21.6) upon stimulation with 0.1 μg mL^−1^ of TcdA (Figure 2). Using these reference ranges (RR), five out of six patients (Patient A–F) that satisfy the diagnostic criteria for FMF^15^ and carry known pathogenic *MEFV* variants had %ASC above the healthy RR; one (Patient C) had %ASC within the healthy RR (Figure 2; Table 1). Patient E had %ASC above the RR prior to genotyping, suggesting that the assay can also be used as a screening test for FMF. All FMF‐positive controls other than Patient A were in remission on colchicine treatment at the time of sample collection. This suggests that disease activity and colchicine treatment do not impact the assay sensitivity.

**Table 1:** Clinical features, genetic testing results and %ASC speck in FMF‐positive controls, non‐FMF autoinflammatory disease controls and cases with ambiguous genetic findings

Patient	Age	Gender	Clinical features	Treatment at time of sampling	Clinical diagnosis	Genetic testing results	%ASC (RR 7–22)
**FMF positive disease controls**							
A	1	F	Early‐onset periodic fevers	Colchicine	FMF	M694V/M694V	**31.3**
B	25	F	Recurrent fevers, abdominal pain, arthritis	Colchicine	FMF	M694V/M694V	**54.9**
C	47	F	Recurrent fevers and abdominal pain	Colchicine	FMF	V726A/V726A	19.3
D	64	F	Recurrent fevers and abdominal pain	Colchicine Anakinra	FMF	M694I/M694I	**34.6**
E	32	F	Recurrent fevers, abdominal pain, back and rib pain	Colchicine	FMF	M694V/R761H	**79.5**
F	31	F	Recurrent fevers, abdominal pain and joint pain	Colchicine	FMF	M680I/R761H	**27.4**
**Other disease controls**							
G	37	M	Early‐onset periodic fevers	Anakinra	TRAPS	TNFRSF1A;c.236C>T	8.0
H	11	F	Early‐onset neutrophilic cold urticaria, aseptic meningitis and congenital hypopituitarism	Anakinra	CAPS	NLRP3:c.1049C>T	7.7
I	2	M	Early‐onset periodic fevers and cold urticaria	Anakinra	CAPS	NLRP3:c.991A>C	5.4
J	54	F	Juvenile idiopathic arthritis, renal granuloma, interstitial nephritis, corticomedullar fibrosis, liver cirrhosis and hepatic granuloma	Mycophenolate	Blau syndrome	NOD2:c.1149G>T	9.3
**Cases with ambiguous MEFV variants**							
K	24	F	Crohn's disease, psoriasis, arthritis, mouth ulcers, tonsillitis, recurrent fevers, family history of autoimmunity	Adalimumab	Undefined autoimmune syndrome	S11C/‐	7.4
L	26	F	Recurrent abdominal pain and fevers	Colchicine Anakinra	FMF (Tel‐Hashomer criteria)	E148Q/E148Q	**67.0**
M	36	M	Recurrent abdominal pain without abdominal pain Colchicine responsive	Nil	FMF (Tel‐Hashomer criteria)	V726A/‐	**53.8**
N	29	F	Fever, abdominal pain, arthralgia	Colchicine	FMF (Tel‐Hashomer criteria)	V726A/K695R	11.6
O	7	F	Recurrent fevers, flushing, conjunctivitis, abdominal pain and limb pain	Colchicine	FMF (Tel‐Hashomer criteria)	E148Q/‐	9.8
**Case with no molecular diagnosis**							
P	8	M	Recurrent fevers, abdominal pain, high CRP	Prednisolone	SURF	Nil	33.5

Red denotes pathogenic/likely pathogenic variants; Orange denotes variants of uncertain significance. Green denotes benign variants as classified by the InFever Database. Bold values indicate result is outside the reference range.

CAPS, cryopyrin‐associated periodic syndrome; FMF, familial Mediterranean fever; RR, reference range; SURF, syndrome of undefined recurrent fever; TRAPS, TNF receptor‐associated periodic syndrome.

### ASC speck formation with mild TcdA stimulation has high specificity for FMF

We also analysed samples from patients with confirmed molecular diagnoses of other autoinflammatory diseases, including TNF receptor‐associated periodic syndrome (TRAPS; Patient G), cryopyrin‐associated periodic syndrome (CAPS; Patients H and I) and Blau syndrome (NOD2; Patient J). None of these autoinflammatory disease controls had a %ASC outside the healthy RR, indicating that the assay was specific for pyrin‐mediated inflammasome overactivity, such as occurs in FMF (Figure 2).

### ASC speck formation with mild TcdA stimulation to diagnose FMF

We next applied the ASC speck assay to five patients with *MEFV* VUS (Patient K–O; Table 1). Patient K was a 24‐year‐old female with Crohn's disease, psoriasis, arthritis, mouth ulcers, tonsillitis and recurrent fevers with a strong maternal family history of autoimmunity. NGS revealed a heterozygous VUS (S11C/−) in *MEFV*. The variant had mostly non‐pathogenic *in silico* scores with a scaled CADD score (v1.6) of 13, a REVEL (v2021‐05‐03) score of 0.116 and one allele in gnomAD 2.1.1. The %ASC speck within the healthy RR confirmed that the VUS was non‐pathogenic. Patient L, M, N and O all satisfied two of the three major Tel‐Hashomer criteria of recurrent febrile episodes with serositis and colchicine responsiveness for the clinical diagnosis of FMF^15^. Patient L was homozygous for E148Q, which has been reclassified as non‐pathogenic in the Infevers database. However, her positive ASC speck assay indicated overactive pyrin inflammasome pathway and was consistent with her response to colchicine. Patient M was heterozygous for a pathogenic V726A variant. Nevertheless, the %ASC above the healthy RR confirmed overactivation of the pyrin inflammasome. Both Patient N and Patient O had %ASC that was within the RR. Patient N is compound heterozygous for the V726A pathogenic variant and the K695R VUS, which has been associated with arthritis. Patient O was heterozygous for the E148Q *MEFV* variant.

Patient P had an autoinflammatory phenotype with recurrent fevers, abdominal pain and arthralgias that resembled FMF but did not have a molecular diagnosis on NGS. There was high %ASC at baseline, which further increased upon TcdA stimulation (Figure 2). The patient was untreated and had active disease at the time of sample collection. These results led to a therapeutic trial of Anakinra, which resulted in partial clinical improvement. He was subsequently trialled on Humira, which induced a sustained clinical and biochemical remission.

## Discussion

FMF is the most common autoinflammatory disease, with a molecular diagnosis providing mechanism‐based rationale for patient access to precision therapy targeting IL‐1β. Ambiguous results, however, can delay diagnosis and treatment. Here, we describe a rapid assay of *MEFV* gene function that can remove this bottleneck in patient care. The use of cryopreserved PBMCs as a source of primary patient CD14+ monocytes has the added advantage that it can be batched and performed by a centralised laboratory without the need for transfection of the VUS in cell lines. In this study, samples were received from multiple centres in Brisbane, Melbourne and Sydney. We used low concentration TcdA stimulation that is able to discriminate healthy controls and autoinflammatory disease controls from FMF patients without the need for colchicine suppression of ASC speck formation. Importantly, FMF patients had positive ASC speck assay results regardless of their disease activity and colchicine treatment.

Our assay measured TcdA‐induced ASC speck formation upstream of cytokine secretion and pyroptosis, both of which may be triggered by other non‐pyrin‐mediated mechanisms.^10^, ^16^ We used immunofluorescence microscopy to directly visualise the ASC specks as an alternative to flow cytometric assays.^11^, ^12^ Widefield fluorescence microscopes are widely available, easily accessible and do not require a skilled operator to optimise staining protocols and gating parameters. While Wittman et al. previously used confocal microscopy to confirm that the indirect flow cytometry‐based assay was indeed able to detect ASC specks,^11^ further studies with larger sample sizes to directly compare the sensitivity and specificity of ASC speck detection by flow cytometry versus microscopy would be warranted.

The counting of ASC specks within live cells is a time‐consuming, labour‐intensive process that is operator‐dependent. By incorporating a semi‐automated algorithm and a custom GUI, we improved the speed and robustness of the assay, reducing operator dependency and increasing reproducibility. The GUI is freely available for other laboratories to implement with minimal equipment and expertise.

Field testing confirmed the reliability of our semi‐automated ASC speck assay in discriminating between patients carrying pathogenic and non‐pathogenic MEFV variants, as has been reported for flow cytometry‐based assay.^17^ However, the sample size in our study is not large enough to accurately calculate the receiver operating characteristics of the assay compared with other methods.

It identified pyrin overactivity in Patient L, who was homozygous for the E148Q ‘non‐pathogenic’ variant, and Patient M, who was heterozygous for V726A. In both cases, this result predicted their clinical response to colchicine and anakinra. Interestingly, Patient O, who was heterozygous for E148Q, had a negative ASC speck assay result. These results reflect the variable penetrance and phenotypic variability of heterozygous *MEFV* variants, suggesting that other additional factors may impact the activity of the pyrin inflammasome.^18^ Patient P, with a provisional diagnosis of syndrome of undefined recurrent fever (SURF)^19^ had a remarkably high %ASC both at baseline and upon stimulation. He responded to anakinra and did not have any *MEFV* variants upon NGS testing, suggesting the involvement of other as‐yet undiscovered genes in this pathway.

In summary, our ASC speck assay can be easily implemented in a laboratory with minimal equipment, cost and expertise beyond cell culture and basic fluorescence microscopy. There is no need for molecular biology expertise as the assay is performed on primary patient cells, and there is no need to transfect cell lines. The open‐source GUI is user‐friendly and easy to implement.

There are limitations to our study. The sample size in the healthy controls, disease controls and FMF‐positive controls is small. Further validation, preferably in a large independent cohort in another laboratory, will be informative. Additionally, it would also be interesting to study patients with other non‐FMF pyrin‐related diseases, such as PSTPIP1‐associated myeloid‐related proteinemia inflammatory (PAMI)^20^ and pyrin‐associated autoinflammation with neutrophilic dermatosis (PAAND).^21^

## METHODS

### Study setting

Patients with the provisional diagnosis of autoinflammatory disease and *MEFV* VUS were referred via the Clinical Immunogenomics Research Consortium Australasia (CIRCA)^22^ listserv for testing. PBMCs were isolated and cryopreserved for batched analysis. Fifteen million cells are thawed for each run.

### CD14+ monocyte isolation

The EasySep Human CD14 Positive Selection Kit II (StemCell Technologies, Vancouver) was used as per manufacturer's instructions. Enriched CD14+ monocytes are plated in 200 μL RPMI 1640 supplemented with 10% foetal bovine serum (FBS) at a concentration of 1 × 10^6^ mL^−1^ to a total of 2 × 10^5^ cells per well in a 96‐well plate.

### Pyrin inflammasome activation

CD14+ monocytes were incubated with 50 μm of freshly prepared VX‐765 Caspase‐1 Inhibitor (Sigma‐Aldrich, St Louis) for 10 min to inhibit cell death/pyroptosis immediately after plating and then with 0.1 μg mL^−1^ of *Clostridium difficile* toxin A (TcdA) (no.: SML1154; Sigma‐Aldrich, St Louis) for 4 h. Control cells are incubated with the Caspase‐1 inhibitors only. Cells were then deposited on Super Frost Plus microscope slides (Thermo Fisher Scientific, Massachusetts) by a Cytofuge 2 (StatSpin) spun at 93 *g* for 6 min and fixed by incubation with 10% Formalin (Sigma‐Aldrich, St Louis) for 7 min.

### ASC speck staining

Fixed cells are initially permeabilised by sequential incubation with 0.1% Triton X‐100 (no.: X100RS‐5G; Sigma‐Aldrich, St Louis) for 20 min and 100 mM glycine (Sigma‐Aldrich, St Louis) for 10 min. Cells are then washed with phosphate‐buffered saline (PBS) and incubated for a further hour with 2% bovine serum albumin (BSA; Sigma‐Aldrich, St Louis).

Cells are then incubated with mouse anti‐ASC monoclonal antibody (no.: SC‐514414; Santa Cruz Biotechnology) diluted 1:100 in 0.55% BSA for 1 h, washed with PBS and then incubated again with Alexa Fluor 647‐labelled donkey anti‐mouse IgG (no.: 715‐605‐151; Jackson ImmunoResearch) diluted 1:500 in 0.5% BSA for another hour in the dark. Cells are then rewashed with PBS and incubated with 4',6‐diamidino‐2‐phenylinodole (DAPI) (no.: D1306; 1:50 000; Thermo Fisher Scientific) diluted 1:50 000 in 0.5% BSA for 5 min in the dark. Excess reagents are discarded by tilting the microscope glass slide and then cover slipped with FluoroMount‐G Media (Thermo Fisher Scientific). Images were then captured using the Leica DM500 microscope using a 20× 0.50 N.A. objective with a 1.25 mm FOV of 712 μm × 532 μm at 1392 × 1040 pixel resolution and pixel size of ~0.5 μm.

### Image analysis and semi‐automated cell and ASC speck counting

**Figure 3:**
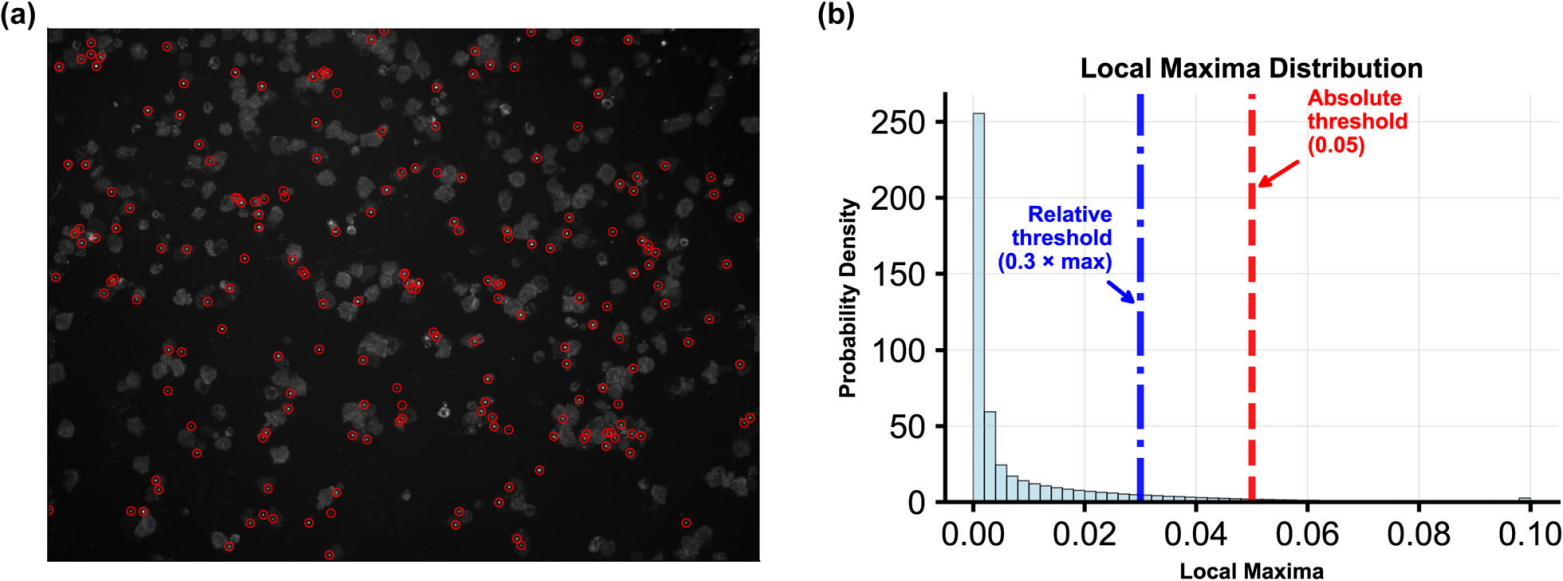
**(a)** Raw image file showing the ASC specks identified by the blob detection algorithm. **(b)** Distribution of local maxima and the thresholds used to filter out inconspicuous puncta.

The GUI programme consists of three main modules. (1) File converter: This module imports raw microscope image files and exports PNG images, which can then be loaded into either Specks Counter or Cells Counter for counting. (2) ASC speck counter: ASC puncta were identified using the blob detection algorithm in scikit‐image,^23^ which employs the Laplacian of Gaussian (LoG) approach to detect puncta of varying sizes and returns their coordinates and approximate radii. Non‐maximum suppression was used to remove overlapping puncta. Absolute and relative thresholds were manually adjusted (whichever was higher) to exclude puncta with local maxima below the defined cut‐offs (Figure 3). (3) Cell counter: This module uses the StarDist^24^ cell segmentation model to detect all live cells in the image. The model was chosen because of its superior performance in discriminating cells of different sizes and shapes.

The programme automatically highlights the boundaries of each live cell and ASC speck, allowing for rapid visual inspection and manual correction of any misidentified objects. For each sample, the final ASC speck and total live cell count were determined by analysing the counts from replicate images taken from five separate regions. On average, we counted 370 cells per image (standard deviation 136). To quantify the degree of pyrin inflammasome activation, the number of intracellular ASC specks was divided by the total number of all live cells (regardless of whether they have intracellular ASC speck), expressed as a percentage of cells with ASC speck (%ASC). We generated a %ASC RR by calculating 95% confidence intervals (CI) on replicate samples from healthy controls (*n* = 10).

### Manual cell and ASC speck counting

For manual counting of cells and ASC specks, microscope image files were opened in ImageJ (V 2.1.90). Cells and intracellular ASC specks were manually counted using the multipoint tool.

### Statistics

Correlation between manual and semi‐automated counts was determined with linear regression, with R^2^ determined by Pearson's correlation coefficient. All calculations were performed in GraphPad Prism 10.

## Author contributions

**Pei Dai:** Conceptualization; data curation; formal analysis; investigation; methodology; project administration; software; validation; visualization; writing – original draft; writing – review and editing. **Oliver Skinner:** Resources; software; visualization; writing – review and editing. **Xufeng Lin:** Conceptualization; data curation; formal analysis; investigation; resources; software; visualization; writing – review and editing. **Aiden Telfser:** Investigation; visualization; writing – review and editing. **Stephanie Ruiz‐Diaz:** Investigation; writing – original draft. **Rohit G Saldanha:** Investigation; resources; writing – review and editing. **Katie Frith:** Investigation; resources; writing – review and editing. **Ming‐Wei Lin:** Investigation; resources; writing – review and editing. **Kahn Preece:** Investigation; resources; writing – review and editing. **Paul E Gray:** Methodology; resources; writing – review and editing. **Alberto Pinzon‐Charry:** Investigation; resources; writing – review and editing. **Anna Sullivan:** Investigation; resources; writing – review and editing. **Stephen Adelstein:** Investigation; resources; writing – review and editing. **Winnie WY Tong:** Investigation; resources; writing – review and editing. **Matthew JS Parker:** Methodology; resources; writing – review and editing. **Laila Girgis:** Investigation; resources; writing – review and editing. **Brynn Wainstein:** Investigation; resources; writing – review and editing. **Samar Ojami:** Investigation; resources; writing – review and editing. **Elissa K Deenick:** Investigation; supervision; writing – review and editing. **Leonard D Goldstein:** Data curation; methodology; resources; software; supervision; writing – review and editing. **Michael J Rogers:** Conceptualization; methodology; supervision; writing – review and editing. **Tri Giang Phan:** Conceptualization; formal analysis; funding acquisition; project administration; resources; supervision; validation; visualization; writing – original draft; writing – review and editing.

## Conflict of interest

The authors declare there are no conflicts of interest.

## Data Availability

All data are available upon request. The open‐source code for the GUI programme is available at https://github.com/lxfhfut/CCT.

## References

[1] The French FMFC , Bernot A , Clepet C *et al*. A candidate gene for familial Mediterranean fever. Nat Genet 1997; 17: 25–31. 9288094 10.1038/ng0997-25

[2] Richards N , Schaner P , Diaz A *et al*. Interaction between pyrin and the apoptotic speck protein (ASC) modulates ASC‐induced apoptosis. J Biol Chem 2001; 276: 39320–39329. 11498534 10.1074/jbc.M104730200

[3] Chae JJ , Wood G , Masters SL *et al*. The B30.2 domain of pyrin, the familial Mediterranean fever protein, interacts directly with caspase‐1 to modulate IL‐1beta production. Proc Natl Acad Sci USA 2006; 103: 9982–9987. 16785446 10.1073/pnas.0602081103 PMC1479864

[4] Gao W , Yang J , Liu W , Wang Y , Shao F . Site‐specific phosphorylation and microtubule dynamics control pyrin inflammasome activation. Proc Natl Acad Sci USA 2016; 113: E4857–E4866. 27482109 10.1073/pnas.1601700113 PMC4995971

[5] Xu H , Yang J , Gao W *et al*. Innate immune sensing of bacterial modifications of rho GTPases by the pyrin inflammasome. Nature 2014; 513: 237–241. 24919149 10.1038/nature13449

[6] Magnotti F , Malsot T , Georgin‐Lavialle S *et al*. Fast diagnostic test for familial Mediterranean fever based on a kinase inhibitor. Ann Rheum Dis 2021; 80: 128–132. 33037005 10.1136/annrheumdis-2020-218366

[7] Van Gorp H , Huang L , Saavedra P *et al*. Blood‐based test for diagnosis and functional subtyping of familial Mediterranean fever. Ann Rheum Dis 2020; 79: 960–968. 32312770 10.1136/annrheumdis-2019-216701 PMC7307214

[8] Shiba T , Tanaka T , Ida H *et al*. Functional evaluation of the pathological significance of MEFV variants using induced pluripotent stem cell‐derived macrophages. J Allergy Clin Immunol 2019; 144: e1412. 10.1016/j.jaci.2019.07.039 31542286

[9] Lu A , Magupalli VG , Ruan J *et al*. Unified polymerization mechanism for the assembly of ASC‐dependent inflammasomes. Cell 2014; 156: 1193–1206. 24630722 10.1016/j.cell.2014.02.008 PMC4000066

[10] Sester DP , Zamoshnikova A , Thygesen SJ *et al*. Assessment of Inflammasome formation by flow cytometry. Curr Protoc Immunol 2016; 114: 14.40.1–14.40.29. 10.1002/cpim.13 27479658

[11] Wittmann N , Behrendt AK , Mishra N , Bossaller L , Meyer‐Bahlburg A . Instructions for flow cytometric detection of ASC specks as a readout of Inflammasome activation in human blood. Cells 2021; 10: 1–16. 10.3390/cells10112880 PMC8616555 34831104

[12] Coudereau R , Gossez M , Py BF *et al*. Monitoring NLRP3 Inflammasome activation and exhaustion in clinical samples: a refined flow cytometry protocol for ASC speck formation measurement directly in whole blood after *ex vivo* stimulation. Cells 2022; 11: 3306. 36291172 10.3390/cells11203306 PMC9600366

[13] Hoss F , Rolfes V , Davanso MR , Braga TT , Franklin BS . Detection of ASC speck formation by flow cytometry and chemical cross‐linking. Methods Mol Biol 2018; 1714: 149–165. 29177861 10.1007/978-1-4939-7519-8_10

[14] Jenster L , Ribeiro LS , Franklin BS , Bertheloot D . Measuring NLR Oligomerization II: detection of ASC speck formation by confocal microscopy and immunofluorescence. Methods Mol Biol 2023; 2696: 73–92. 37578716 10.1007/978-1-0716-3350-2_5

[15] Livneh A , Langevitz P , Zemer D *et al*. Criteria for the diagnosis of familial Mediterranean fever. Arthritis Rheum 1997; 40: 1879–1885. 9336425 10.1002/art.1780401023

[16] Netea MG , Simon A , van de Veerdonk F , Kullberg BJ , van der Meer JW , Joosten LA . IL‐1beta processing in host defense: beyond the inflammasomes. PLoS Pathog 2010; 6: e1000661. 20195505 10.1371/journal.ppat.1000661 PMC2829053

[17] Honda Y , Maeda Y , Izawa K *et al*. Rapid flow cytometry‐based assay for the functional classification of MEFV variants. J Clin Immunol 2021; 41: 1187–1197. 33733382 10.1007/s10875-021-01021-7

[18] Aksentijevich I , Schnappauf O . Molecular mechanisms of phenotypic variability in monogenic autoinflammatory diseases. Nat Rev Rheumatol 2021; 17: 405–425. 34035534 10.1038/s41584-021-00614-1

[19] Papa R , Penco F , Volpi S , Sutera D , Caorsi R , Gattorno M . Syndrome of undifferentiated recurrent fever (SURF): an emerging Group of autoinflammatory recurrent fevers. J Clin Med 2021; 10: 1963. 34063710 10.3390/jcm10091963 PMC8124817

[20] Dai P , Furlong T , Gracie G *et al*. Autoinflammation masquerading as autoimmunity in an adult with heterozygous p.E250K PSTPIP1 mutation. J Clin Immunol 2019; 39: 519–522. 31119601 10.1007/s10875-019-00646-z

[21] Schnappauf O , Chae JJ , Kastner DL , Aksentijevich I . The pyrin Inflammasome in health and disease. Front Immunol 2019; 10: 1745. 31456795 10.3389/fimmu.2019.01745 PMC6698799

[22] Phan TG , Gray PE , Wong M , Macintosh R , Burnett L , Tangye SG . The clinical Immunogenomics research consortium Australasia (CIRCA): a distributed network model for genomic healthcare delivery. J Clin Immunol 2020; 40: 763–766. 32483663 10.1007/s10875-020-00787-6

[23] van der Walt S , Schonberger JL , Nunez‐Iglesias J *et al*. scikit‐image: image processing in python. PeerJ 2014; 2: e453. 25024921 10.7717/peerj.453 PMC4081273

[24] Schmidt U , Weigert M , Broaddus C , Myers G . Cell detection with star‐convex polygons. In: Frangi AF , Schnabel JA , Davatzikos C , Alberola‐López C , Fichtinger G , eds. Medical Image Computing and Computer Assisted Intervention – MICCAI 2018. Cham: Springer International Publishing; 2018:265–273.

